# Indoor air pollution from firewood combustion in Indigenous *malocas* in the Brazilian Amazon: exposure to fine particulate matter and associated health risks

**DOI:** 10.36416/1806-3756/e20250161

**Published:** 2025-09-22

**Authors:** Adriana Gioda

**Affiliations:** 1. Departamento de Química, Pontifícia Universidade Católica do Rio de Janeiro, Rio de Janeiro (RJ) Brasil.

Firewood remains a primary source of domestic energy for millions of people worldwide, especially those in low- and middle-income countries. In Brazil, this reality is particularly acute among Indigenous, *quilombola*, and riverine populations living in remote areas with limited access to electricity or liquefied petroleum gas.^1^ Although the use of biomass fuel is deeply rooted in cultural traditions, it also presents a serious and often overlooked public health concern: chronic exposure to indoor air pollution, especially fine particulate matter, i.e., particles that are equal to or less than 2.5 µm in diameter (PM_2.5_). 

In Brazil, there are approximately 1.7 million Indigenous people, most of whom face vulnerabilities such as poverty, limited health care access, and energy insecurity. This letter presents findings from a pilot study conducted in traditional Indigenous dwellings (*malocas*) across various ethnic communities in the Legal Amazon. The study sought to quantify PM_2.5_ concentrations from firewood combustion and assess associated noncarcinogenic health risks. Nine villages were visited, each including 1-20 *malocas*. Approximately 80% were sampled during medical outreach, with sampling durations ranging from 20 min to 1 h. The fire was not lit in some huts, which were therefore compared with those in which it was. Additionally, outdoor measurements were taken in order to identify the sources of PM_2.5_. Because of family challenges and the lack of electricity, the sampling time could not be extended. A daily exposure of 8 h was assumed on the basis of observations that Indigenous individuals, especially women and children, spend an average of 8 h near indoor fire pits. Each *maloca* typically housed five to seven occupants from extended families. Although the primary objective of the pilot study was to characterize exposure levels, preliminary discussions were initiated with community members and leaders regarding potential mitigation strategies, such as improving ventilation and modifying the design of fire pits. An air quality monitor with a PM_2.5_ sensor of 0-999 µg • m^-3^ (TEMTOP M-2000, Elitech, Brazil) was used in order to measure PM_2.5_ levels, as reported elsewhere.^1^

The results show that PM_2.5_ concentrations varied significantly between the indoor environment and the outdoor environment. The mean indoor PM_2.5_ concentration during the burning of firewood was 203 ± 261 µg/m^3^ (range, 20-999 µg/m^3^), whereas outdoor levels averaged only 9.5 ± 5.5 µg/m^3^ (range, 0.7-23 µg/m^3^). Indoor concentrations exceeded the WHO daily recommendation of 15 µg/m^3^ by more than tenfold, whereas outdoor levels remained within the recommended limit. Indoor-to-outdoor ratios > 1 confirmed that indoor combustion was the primary source of PM_2.5_. Significant differences (p < 0.05) were observed between the following scenarios: fire on vs. fire off; fire on vs. liquefied petroleum gas; and indoor vs. outdoor environments. These findings highlight the critical role of combustion type and ventilation in shaping exposure. 

Other studies involving Indigenous communities reported high indoor concentrations of PM_2.5_. Bunnell et al.^2^ reported mean indoor PM_2.5_ levels of 38 µg/m^3^ among Navajo homes using coal-fired heating. In comparison, the Hopi tribe showed mean concentrations of 36.2 µg/m^3^, which decreased to 14.6 µg/m^3^ without heating.^3^ Although these values are concerning, they remain substantially lower than those observed in the *malocas* in Brazil, where open fires are used daily for cooking and warmth. In Mexico, Hernández et al.^4^ documented a mean indoor PM_2.5_ concentration of 114 ± 140 µg/m^3^ among the Tzotzil. In Bolivia, Quechua homes reached daily averages of 240 ± 210 µg/m^3^.^5^ The aforementioned studies suggest that PM_2.5_ levels in homes relying on biomass can vary greatly on the basis of fuel type, duration of exposure, housing structure, and cooking practices. 

To estimate noncarcinogenic risk, we applied the U.S. Environmental Protection Agency hazard quotient model.^6^ The hazard quotient is calculated as the ratio of average daily dose to reference dose. For PM_2.5_, the reference dose used was 5 µg/kg/day. Using a measured indoor concentration of 203 µg/m^3^, we applied the following equation:

ADD=C×IR×EF×ED/BW×AT

where *ADD* is the average daily dose; *C* is the indoor concentration; *IR* is the inhalation rate (20 m^3^/day); *EF* is the exposure frequency (8 h/day, i.e., 0.333 days/day); *ED* is the exposure duration (30 years); *BW* is the body weight (70 kg); and *AT* is the average time (30 × 365 = 10,950 days). 

The resulting hazard quotient was 3.84, which was significantly above the threshold of 1, indicating a potential for chronic adverse effects such as respiratory and cardiovascular disease. 

To our knowledge, the pilot study reported herein represents the first quantitative assessment of PM_2.5_ exposure in Indigenous Brazilian *malocas*. Although the study acknowledges limitations related to sampling duration and other factors, it underscores the urgent need for further studies aimed at understanding the health risks associated with biomass combustion. In a previous study, we found high PM_2.5_ levels attributed to open-fire wood burning, a finding suggesting that similar conditions are present in the *malocas*.^1^ Indoor sources of PM_2.5_ can contribute to respiratory diseases and deaths. Data from the Brazilian National Ministry of Health Special Department of Indigenous Health show that 21.6% of all deaths among Indigenous children under one year of age are attributed to respiratory illnesses, underscoring a preventable health crisis. Furthermore, 2024 data from the Brazilian *Núcleo Ciência Pela Infância* show that respiratory diseases are the leading cause of death among Indigenous children under four years of age, accounting for 18% of all deaths in this age group. In Guarani communities, overcrowded homes with open fires have been strongly linked to higher hospitalization rates for respiratory infections.^7^

To safeguard these communities, it is essential to strengthen Indigenous health systems; invest in household energy transitions; and conduct long-term exposure assessments. 
